# Explosive property and combustion kinetics of grain dust with different particle sizes

**DOI:** 10.1016/j.heliyon.2020.e03457

**Published:** 2020-03-04

**Authors:** JiangPing Zhao, GongFan Tang, YaChao Wang, Yujiu Han

**Affiliations:** School of Resource Engineering, Xi'an University of Architecture & Technology, Xi'an, 710055, PR China

**Keywords:** Energy, Materials chemistry, Biofuel, Biomass, Energy sustainability, Materials characterization, Materials safety, Explosion, Combustion kinetics, Heat release properties, Coats-redfern integral method, Particle size, Hartmann tube

## Abstract

The effect of particle size on the combustion and explosion properties of grain dust is investigated by Hartmann tube, cone calorimeter (CC), and thermogravimetry (TG), it aims to provide fundamental experimental data of grain dust for an in-depth study on its potential risk. The fine-grain dust facilitates the decrease in the minimum ignition temperature (MIT) of dust layer and dust cloud, as well as the obvious increases in the maximum explosion pressure *P*_max_ (climbs from 0.36 to 0.49 MPa) and pressure rising rate d*P*/dt (rises from 6.05 to 12.12 MPa s^−1^), leading to the increases in maximum combustion rate (*dw/dτ*)_max_ and combustion characteristic index *S*, corresponding to the greater or severer potential risk. Because the *E* corresponding to combustion increases from 106.05 (sample with a particle size of 180–1250 μm) to 153.45 kJ mol^−1^ for the sample of 80–96 μm, the combustion process gradually transforms from diffusion-controlled into a kinetically controlled mode with the decreasing particle size of grain dust, together with the retardation of initially transient charring. It determines that the competition between the charring and combustion dominates the decomposition, and the combustion prevails for the coarse particle, while the charring controls the combustion for the fine-grain dust.

## 1 Introduction

With the depleting fossil fuel resources, increasing environmental concerns, and political commitment, sustainable development has been a highly multi-disciplinary field [1], the recent two decades have already witnessed the booming development of biomass fuel feedstock, which has been employed as an alternative to the diminishing coal. Although many drawbacks limit its extensive applications, the major environmental, economic, and social benefits appear to compensate for the technological and other barriers caused by its unfavorable composition and properties of biomass fuel [2]. Therefore, continuously increasing attention has been paid on maize grain due to its ecological hotspot based on “business as usual” conventional farming practice [3]. Additionally, cereal residues as renewable and abundant resources, include both on-site residues and processing residues, have a huge potential to achieve more sustainable agriculture and to provide a novel fuel feedstock in theory [4]. However, the suspending grain dust in production departments is detrimental to life safety during liquor-making and starch processing, which holds potential hazard of fire or explosion due to its flammability and low density for forming an explosive cloud [5, 6, 7]. The necessary prevention and control of organic dust explosion are very imperative for safety production and property security, and the quantitative research on the potential explosion property is the prerequisite to designing some effective and efficient safeguards to minimize its security risk.

Consequently, the quantitative analysis on the combustion and explosion properties of organic powders have attracted increasing interest, the particle size has become a breakthrough point to prompt the suppression design. Castellanos et al. [8] have analyzed the thermal stability of cornstarch with phosphates and determined the crucial role of particle size on improving the inhibiting rate of heat absorption. Yu et al. [9] have investigated ammonium polyphosphate on explosion characteristics of micron-size acrylates copolymer powders. Addai et al. [10] have compared the MIT of five different dust and six different gases. The combustion of wheat starch and carbon-black particles have also been studied [11]. Addai et al. [12] also assert that the inert materials with high bulk density are not ideal inertants. Generally, the devolatilization and char oxidation mainly predominate the whole combustion process [13], seeking an appropriate inertant for explosion suppression of organic dust is intriguing the increasing attention, but a few reports focus on the explosion property of organic dust.

Furthermore, biomass dust has also shown strong flammability, it holds a high-explosive tendency and easily transforms into the hazard, although it does not occur in the same way as in the case of coal. The different sizes and various shapes of organic powder make the interpretation of organic dust explosion difficult and complicated [14, 15]. Saeed et al. [16, 17] suggest that fine biomass facilitates increased mass-burning with high flame speed. Therefore, the potential risk derived from the organic dust including explosion and ignition imparts huge hidden danger to the dust-processing workshops [5, 6, 7]. The preliminary exploration of the ordinary or pristine organic dust is necessary to broaden the combustion and explosion mechanism, which is beneficial to provide some basic experimental data in the security design of dust explosion suppression.

However, there is scant data on the combustion kinetics and explosion characteristics of grain dust to promote comprehensive research. Consequently, using the grain powder of beer production workshop as the research objective to approach realistic scenario, the effect of particle size on the minimum ignition temperature (MIT) is measured based on the experimental dust layer and dust cloud, the lower explosive limit (LEL), heat-release properties, and dynamic characteristics are tested by the Hartmann tube, cone calorimeter, and thermal gravimetry (TG), respectively. It aims to provide an exploration on the combustion and explosion properties of grain dust, prompting its recycling as a novel fuel feedstock and diminishing its security risks. Additionally, the combustion mechanism of biomass is in its beginning stage, the quantitative analysis of grain powder is lacking, the Coats-Redfern integral method is employed to illustrate the combustion kinetics firstly. The article's novelty relates to combining the calculation of combustion characteristics with combustion kinetics simultaneously, it establishes an effective quantitative analysis method on dust-explosion of organic powder.

## 2 Materials and methods

### 2.1 Raw materials

**Figure 1:**
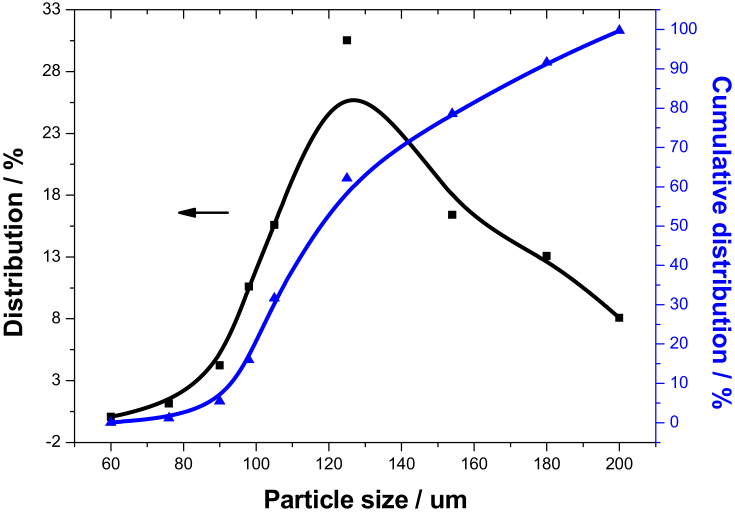
Size distribution of pristine grain dust.

The pristine powder consisted of grain and chaff dust was collected from the dust removal system of Xi'an brewery in Shaan'xi province of China, it mainly was composed of malt dust, rice dust, and a small amount of ash as shown in Table 1, presented the features of high volatility, poor LHV, and low fixed carbon. The grain dust sample with different particle size was sieved by the standard sieve, which was obtained by sieving of 80 mesh (180–1250 μm), 100 mesh (154–180 μm), 120 mesh (120–154 μm), 140 mesh (109–120 μm), 160 mesh (109–96 μm), and 180 mesh (80–96 μm), respectively. The distribution curve of pristine grain dust was drawn by Gaussian fitting in Figure 1, the sieve residues were served as the research objective.

**Table 1:** Proximate and ultimate analysis of pristine grain dust.

Elemental analysis/%						Industrial analysis (drying)				
Element	C	H	O	N	Other	Fixed carbon/%	Moisture/%	Ash/%	Volatile/%	LHV/(MJ.kg^−1^)
Content	43.82	5.83	43.36	2.48	4.51	13.86	7.76	5.43	72.95	16.19

Note: LHV denotes the lower heating value.

### 2.2 Characterizations

The MIT of the grain dust layer with a thickness of 5 mm and dust cloud was recorded by the testers made in Northeast University of China, according to the standards of GB/T 16430-1996 and GB/T 16429-1996, respectively. The LEL concentration, explosion pressure rise rate d*P*/dt, and the maximum explosion pressure *P*_max_ were measured by a 1.2 L Hartmann tube. The affiliated electronic data acquisition system was employed to record the real-time explosive parameters, which could transform the pressure into a voltage value to obtain real-timely explosive parameters. The experimental condition was conducted at 29 °C with an ignition delay time of 8 s, the spraying-powder pressure was 0.5–0.6 MPa with an error of 0.01 MPa for *P*_max_.

The cone calorimeter (CC, ZY6243, Zhongnuo instrument company, China) was exploited to record the real-time heat release property according to ISO-5660-1-2015. Each specimen consisted of 9 symmetrically cylindrical pancakes with a diameter of 2 cm and a height of 3 mm, which were fabricated by the manually hydraulic-forming press with a pressure of 0.3 MPa and wrapped in aluminum foil. The heat release parameters of samples were calculated from the average of 3 determinations with a standard deviation < 10%, under an external heat flux of 35 kW m^−2^ (600 °C approximately), including the time to ignite (TTI), the peak heat release rate (p-HRR), and the time to p-HRR (t_p_). Besides, the two important indexes of fire performance index (FPI, FPI = TTI/p-HRR) and the fire growth index (FGI, FGI = p-HRR/t_p_) were used to evaluate the combustion performance. Furthermore, non-isothermal combustion kinetics was calculated by a TG analyzer (Mettler, Germany) under simulated air during the heating process of 50–600 °C with a heating rate of 20 °C·min^−1^.

## 3 Result and analysis

### 3.1 MIT

Table 2 discloses that the MIT of dust layer/cloud sample declines with the decreasing size, which facilitates the diffusion and permeation of oxygen, leading to a decrease in MIT. However, the MIT maintains the same when the particle size (160 mesh) reduces further, indicates that the combustion is not only controlled by oxygen concentration, although the fine dust could trap and accommodate more O_2_ from the air. Meanwhile, the MIT of the dust cloud is far above that of the dust layer, it might be ascribed to the lower heat conductivity of air, the suspending particles in the vessel as the dust cloud, resulting in a slow rise of dust surface temperature.

**Table 2:** MIT of samples and raw material.

Samples	Shape	Raw material	80 mesh	100 mesh	120 mesh	140 mesh	160 mesh	180 mesh
MIT/°C	Cloud	480 ± 2	490 ± 2	470 ± 2	450 ± 2	440 ± 2	430 ± 2	430 ± 2
	Layer	135 ± 2	140 ± 2	135 ± 2	130 ± 2	130 ± 2	130 ± 2	130 ± 2

### 3.2 Explosion property tested by Hartmann tube

The subtle change on the maximum explosion pressure *P*_max_ is detected, but the pressure rising rate d*P*/dt drop with the increasing particle size as shown in Table 3, while the LEL rises, indicates that the reactivity of grain dust weakens with the increasing particle size. Since the enhancement of specific surface area favors the permeation of O_2_, together with the higher surface energy due to the fine dust. It leads to the rising possibility of deflagration evidenced by the increases in the *P*_max_ and d*P*/dt. It is consistent with the finding that minimum ignition energy tends to have lower values as the particle size decreases [18, 19]. According to the mechanism of “pyrolysis-devolatilization” [13, 20], the decrease of particle size accelerates the pyrolysis of hemicellulose and forms more content of combustible gas. When the fine dust only consists of C, H, O, and N, it will be vaporized before the flame front reaches, and forms well mixed O_2_-containing fuel gas, which promotes the occurrence of dust explosion. However, the inorganic minerals in grain particles alter the process by forming a boundary or barrier, resulting in that vaporization doesn't finish before the flame front reaches. As a consequence, the grain dust is partially vaporized and forms locally fuel-rich gas, leading to the weakened explosion.

**Table 3:** Explosion parameters of grain dust sample.

Samples	LEL/g·m^−3^	*P*_max_/MPa	d*P*/dt/MPa·s^−1^
180 mesh	50∼58.33	0.49 ± 0.01	12.15 ± 0.2
160 mesh	58.33∼66.67	0.45 ± 0.01	9.35 ± 0.2
140 mesh	64∼71.48	0.43 ± 0.01	8.73 ± 0.2
120 mesh	75∼83.33	0.42 ± 0.01	7.33 ± 0.1
100 mesh	116.67∼125	0.41 ± 0.01	7.28 ± 0.1
80 mesh	141.67∼150	0.36 ± 0.01	6.05 ± 0.1

### 3.3 Heat release property

**Figure 2:**
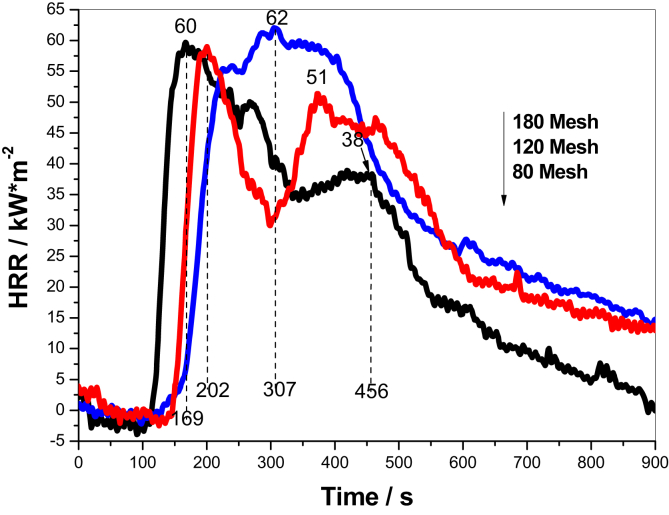
HRR of grain dust with different size.

The heat release rate of grain dust presents two peaks as shown in Figure 2, the first peak is attributed to gases and volatiles derived from the rapid decomposition of hemicellulose [21], simultaneous charring layer shields the flame gradually, leading to a slight decrease in HRR. And then the initial char cracks and triggers the second huge peak under the continuous heating condition, which involves unburned grain dust, cellulose and lignin left. Finally, a compact char layer covers on the surface of the sample rather than the afterglow phenomenon is observed after burning in CC [22], reveals the occurrence of rapid charring. The feature of a double peak becomes distinct for the dust with a particle size of 120–154 μm. Due to the enhancement of charring, the quickly formed char-layer effectively inhibits the transfer of heat and mass. However, a broad and right-shifted peak appears for the sample with a particle size of 80–96 μm, which might be caused by that the rapid charring covers the grain pancake completely at the beginning of burning, accumulates energy and blocks the permeation of O_2_. However, the incremental volatiles derived from the decomposition of grain dust rush out the superficial char shell and trigger a vigorous flashover, when the accumulated energy surpasses a critical value, presenting a single and broad heat-releasing peak.

The heat release property of grain powder is briefed in Table 4, the THR and weight loss drop with the decreasing particle size, while the TTI and t_p_ increase, due to the enhancement of heat storage capacity and retardation of initially transient charring of 80–96 μm sample, leading to the overlapping of heat release peaks involved in hemicellulose and cellulose. Additionally, the increased FPI and decreased FGI are assigned an enhanced flame-resistant efficiency for the sample with a particle size of 80–96 μm [23]. However, the value of p-HRR exhibits insignificant changes, infers that the maximum flame radiant intensity of fine dust remains the same as that of the coarse particle.

**Table 4:** Heat release properties of grain powder with different particle sizes.

Samples	THR/kJ	TTI/s	t_p_/s	p-HRR/kJ·m^−2^	FPI/s ·m^2^ kW^−1^	FGI/kW·m^−2^ ·s^−1^
80 mesh	376.62 ± 7	69 ± 1	169 ± 1	60.67 ± 2	1.14	0.36
120 mesh	344.47 ± 7	83 ± 1	202 ± 1	60.21 ± 2	1.38	0.30
180 mesh	307.89 ± 6	93 ± 1	307 ± 1	61.76 ± 2	1.51	0.20

### 3.4 Combustion characteristics and kinetics

#### 3.4.1 Combustion characteristics

The combustion characteristics of samples contain ignite index *D*_*i*_ [24, 25] and comprehensive combustive characteristic index *S* [26], which are calculated by formulas (1), (2), (3), respectively.

(1) $$D_{i}=\frac{{\left(dw/d\tau \right)}_{\max}}{T_{i}\cdot T_{p}}$$

The (*dw/dτ*)_max_ is the maximum combustion rate, which also is denoted as DTG_max_, %·min^−1^; T_i_ and T_p_ determined by TG/DTG method are assigned to ignition and peak temperature respectively, K.

(2) $$S=\frac{{{\left(dw/d\tau \right)}_{\max}\left(dw/d\tau \right)}_{mean}}{T_{i}^{2}T_{f}}$$

The (dw/dτ)_mean_ is calculated by the formula (3) corresponding to the average burning rate, τis the time, min; T_f_ is the burnout temperature with a weight loss of 95%, K.

(3) $${\left(dw/d\tau \right)}_{mean}=\frac{wl}{\left(T_{f}-T_{0}\right)/V_{T}}$$

The *wl* in formula (3) equals the ratio of the deviation to the starting weight, the deviation is the weight change between the starting weight and the final weight of samples after heating from 50 °C to 600 °C. The T_0_ is the beginning heating temperature of 323 K, *V*_T_ is the heating rate of 20 °C min^−1^.

The T_i_, *S*, (dw/dτ)_mean_, DTG_max_, and *D*_*i*_ rise with the decreasing particle size in Table 5, implies the intensive possibility of deflagration, and the DTG_max_ is dramatically improved, which increases from 9.47 to 16.63 %·min^−1^. Combining with the retardant T_i_ and lessened T_f_, it verifies the occurrence of violent combustion for the fine dust of 80–96 μm.

**Table 5:** Combustion characteristic parameters of grain dust with different sizes.

Samples	T_i_/K	T_p_/K	T_f_/K	DTG_max_/%·min^−1^	*S* (×10^−7^)	*D*_*i*_(×10^−5^)	(dw/dτ)_mean_ %·min^−1^
80 mesh	520 ± 2	574 ± 1	746 ± 2	9.47	1.52	3.17	3.23
120 mesh	533 ± 2	581 ± 1	747 ± 2	11.22	1.74	3.62	3.29
180 mesh	550 ± 2	583 ± 1	738 ± 2	16.63	2.50	5.19	3.36

#### 3.4.2 Combustion kinetics

**Figure 3:**
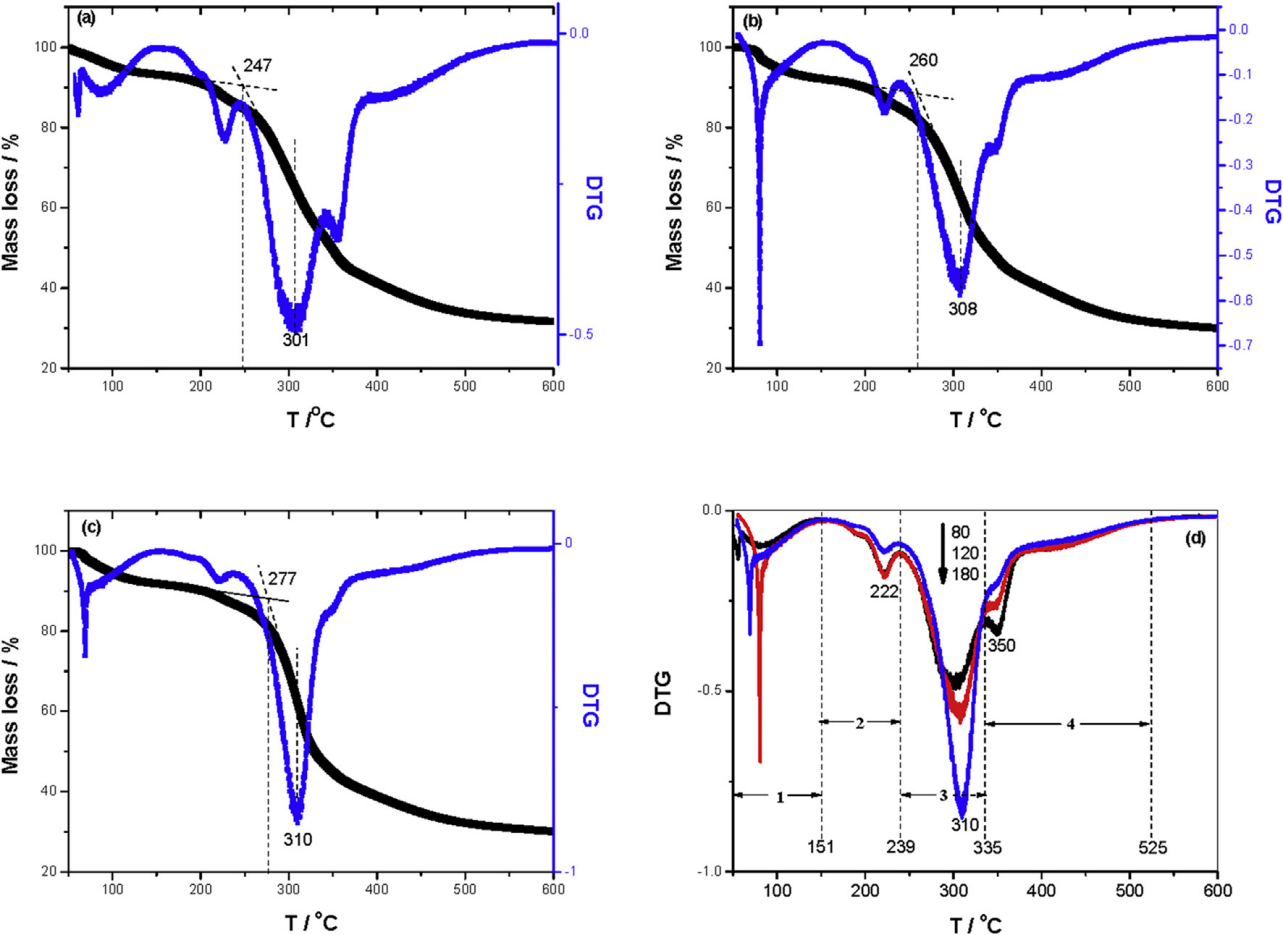
TG/DTG of grain dust with different size including (a)80 mesh, (b)120 mesh, (c)180 mesh, and (d) DTG.

According to the references [27, 28], the combustion process of grain dust could be divided into the following four stages according to the DTG curves in Figure 3: moisture evaporation (50–151 °C), pyrolysis and combustion of hemicellulose (151–239 °C), pyrolysis and combustion of cellulose (239–335 °C), as well as pyrolysis and combustion of lignin (335–525 °C). Since the low lignin content in the sample (about 15% [29]), the weight loss peak corresponding to the lignin is almost absent. Because cellulose is a high-molecular compound with long linear chains composed of D-glucosyl group, partial cellulose has a crystalline structure made of ordered microfibrils, results in thermal degradation is more difficult than hemicellulose [27]. The higher capacity of heat storage derived from the decreasing particle size causes the overlapping of DTG curves of hemicellulose and cellulose, presents the diminishing peak of hemicellulose and lignin while the enhanced peak of cellulose, which is in agreement with the results of HRR.

The combustion dynamics of samples are analyzed by formulas (4), (5), (6), according to the Coats-Redfern integral method with the first-order reaction, taking the value of R^2^ into account, the results are listed in Table 6.

(4) $${f\left(\alpha \right)=\left(1-\alpha \right)}^{n}$$

(5) $$\frac{d\alpha }{dT}=\frac{A}{\beta }\exp\left(-\frac{E}{RT}\right)f\left(\alpha \right)$$

(6) $$\ln\left[\frac{G\left(\alpha \right)}{T^{2}}\right]=\ln\left(\frac{AR}{\beta E}\right)-\frac{E}{RT}$$

**Table 6:** Combustion kinetic parameters of samples with different sizes.

Samples	Temperature	*E*/kJ·mol^−1^	lg A/s^−1^	Adj. R^2^
80 mesh	∼188.7 °C	4.41	-2.9	0.99
	188.7∼226.9 °C	44.4	0	0.95
	226.9 °C∼282.4 °C	60.12	2.5	0.95
	282.4 °C∼360.1 °C	106.05	10.5	0.98
	360.1 °C∼537.3 °C	11.43	0	0.84
120 mesh	∼188.7 °C	6.36	-3.1	0.99
	188.7∼226.9 °C	36.36	0	0.92
	226.9 °C∼282.4 °C	55.65	2.2	0.99
	282.4 °C∼360.1 °C	119.58	11.7	0.99
	360.1 °C∼537.3 °C	34.92	0	0.99
180 mesh	∼188.7 °C	2.85	-3.1	0.99
	188.7∼226.9 °C	18.93	0	0.87
	226.9 °C∼282.4 °C	45.84	0	0.95
	282.4 °C∼360.1 °C	153.45	10.3	0.99
	360.1 °C∼537.4 °C	10.23	0	0.89

The weight loss rate α is calculated by the equation of α=(m_0_-m_t_)/(m_0_-m_f_) (m_0_ is the starting weight, m_t_ is the real-time dust weight, and m_f_ denotes the finally remaining weight). The n is the reaction order as 1, 2, 3…. A is the pre-exponential factor, min^−1^; *E* is the activation energy, kJ·mol^−1^. *R* is the universal gas constant, kJ·(mol·K)^−1^. *β* is the heating velocity of 20 °C min^−1^. T is the absolute temperature, K. The G(α) is calculated by the equation of$G\left(\alpha \right)=\int_{0}^{\alpha }\frac{d\alpha }{f\left(\alpha \right)}$, and the A, E, and n are achieved by plotting ln [G(α)/T^2^] against 1/T.

Since the ignition temperature of all samples ranges from 240 °C to 270 °C, its combustion mechanism appertains to static permeable diffusion combustion, the burning process begins with the ignition. The combustion reaction of grain dust mainly takes place during 282.4–360.1 °C, and the linear correlation coefficients of the fitting curves are in close proximity to1. The reaction activation energy *E* corresponding combustion increases from 106.05 (180–1250 μm sample) to 153.45 kJ mol^−1^, it reveals the higher energy barrier to combustion for the fine sample than that of the coarse particle, the combustion gradually transforms from diffusion-controlled into kinetically controlled reaction with the decreasing particle size.

## 4 Discussion

It is well known that grain powder contains inorganic elements as sodium, potassium, calcium, and silicates, the mineral matter may prompt an enhanced carbonaceous layer on the dust surface that restricts the oxygen access and retards the ignition process [5, 30]. Because the sodium and potassium involved in the grain dust lower the melting point of ash [31, 32, 33, 34], and a certain amount of SiO_2_ triggers slagging and prompts the formation of a charring shell [35], leading to blocking effect to combustion-flame propagation and increases in ignition temperature and MIT.

According to the above experimental data analysis, the following mechanism could be obtained. The combustible volatiles in grain dust absorb heat and transform into combustible gas under the heating firstly, which could react with O_2_ and generate flame; and then partially superficial dust transforms into carbonaceous shielding-layer, while the inner combustible gas derived from the pyrolysis of combustible volatile diffuses continuously towards the surface, the particle is quickly enveloped in flames. Therefore, due to the enhancement of heat storage capacity, the abrupt release of accumulated heat intensifies flashover when the volatile pressure surpasses a critical value, leading to the obvious enhancement on the *P*_max_ and d*P*/dt, indirectly evidenced by the increased DTG_max_ for fine-grain dust of 180 mesh. Meanwhile, the rapidly soared temperature of the outer layer facilitates and accelerates the charring of inner grain dust, leading to an increase in the activation energy. Li et al. [36] found that aluminum dust flame speed would increase and the combustion would transform from diffusion-controlled mode to kinetically controlled mode with the decreasing particle size. Apart from the enhancement of *D*_*i*_ and *S* with the decreasing particle size of grain dust, our study determines that the combustion process is firstly accelerated with the increased oxygen concentration, followed by a blocking effect due to the charring with the decreasing particle size, it transforms from diffusion-controlled mode to kinetically controlled mode. Generally, the competition between the charring and combustion dominates the decomposition process of grain dust, and the combustion prevails for the coarse particle, while the charring controls the combustion for the fine dust.

The discrepancy between HRR and combustion characteristic parameter lies in the variant focuses, the TTI and p-HRR are crucial to evaluate the fireproof efficiency, while the DTG_max_ determines the combustion property. That is, the initially transient charring presents a higher flame retardant efficiency, but the followed enhancement of DTG_max_ favors vigorous flashover or deflagration with disastrous risk. Therefore, the HRR hardly match the combustion characteristic, the former focuses on the final result of burning while the latter real-timely supervises the whole burning. Consequently, the combination of the two techniques is recommended to assess the combustion performance effectively.

Furthermore, the reported results show indistinctive changes on the MIT and P_max_ between the highest and the lowest particle size studied. However, the foremost parameter relates to the dust explosion as dP/dt, which holds the potential danger and easily leads to damage, casualties or injuries, clarifying the effect of particle size on the explosion severity of grain dust is prominent important for security design of dust-explosion prevention and control, rather than the further theoretical research. Moreover, a novel quantitative analysis method combined the calculation of combustion characteristics, CC, with the combustion kinetics is proposed. It opens up a comprehensive method to evaluate the explosion of grain dust and extends the method database for risk assessment of ordinary dust-processing.

Although the parameters on combustion and explosion property of grain dust are examined, the microstructure and its chemical bonding need further research in the future. The combustion kinetics preliminarily studies the combustion mechanism, which is confirmed by the results of CC and TG. However, the interactions between the superficial carbonaceous layer and the underlying grain dust, the effect of minor mineral elements involved in grain dust, and the configuration of grain dust hold broad research spaces.

## 5 Conclusions

A preliminary study on the effect of particle size on the combustion and explosion properties of grain dust is investigated to provide some basic experimental data in the security design of dust-explosion prevention and control, the CC and TG are employed to illustrate the mechanisms of combustion kinetics firstly, and the following conclusions are drawn.

(1) The *P*_max_ and d*P*/dt increase from 0.36 to 0.49 MPa and from 6.05 to 12.12 MPa s^−1^, respectively, when the particle size of grain dust decreases from 80 mesh to 180 mesh. It demonstrates that the competition between the charring and combustion dominates the decomposition of grain dust, and the combustion prevails for the coarse particle, while the charring controls the combustion for fine dust.
(2) The *E* of combustion calculated by the Coats-Redfern integral method climbs from 106.05 (180–1250 μm sample) to 153.45 kJ mol^−1^ for the sample with a particle size of 80–96 μm. It reveals that the combustion process transforms from diffusion-controlled into kinetically controlled reaction with the decreasing size of grain dust.
(3) It elucidates that the HRR hardly match the combustion characteristic index tested by TG, ascribe to the different evaluation criterion, the former focuses on the final result of burning while the latter real-timely supervises the whole burning. But the combination of the two techniques is recommended to assess the combustion performance effectively.

## Declarations

### Author contribution statement

JiangPing Zhao: Conceived and designed the experiments; Analyzed and interpreted the data.

GongFan Tang: Performed the experiments; Analyzed and interpreted the data; Contributed reagents, materials, analysis tools or data.

YaChao Wang: Contributed reagents, materials, analysis tools or data; Wrote the paper.

Yujiu Han: Contributed reagents, materials, analysis tools or data.

### Funding statement

The authors sincerely acknowledge the financial support by China Scholarship Council (CSC No. 201808610034).

### Competing interest statement

The authors declare no conflict of interest.

### Additional information

No additional information is available for this paper.
